# Deep learning, radiomics and radiogenomics applications in the digital breast tomosynthesis: a systematic review

**DOI:** 10.1186/s12859-023-05515-6

**Published:** 2023-10-26

**Authors:** Sadam Hussain, Yareth Lafarga-Osuna, Mansoor Ali, Usman Naseem, Masroor Ahmed, Jose Gerardo Tamez-Peña

**Affiliations:** 1 School of Engineering and Sciences, Tecnológico de Monterrey, Ave. Eugenio Garza Sada 2501, 64849 Monterrey, Mexico; 2 College of Science and Engineering, James Cook University, Cairns, Australia; 3 School of Medicine and Health Sciences, Tecnológico de Monterrey, Ave. Eugenio Garza Sada 2501, 64849 Monterrey, Mexico

**Keywords:** Deep learning, Radiomics, Radiogenomics, Digital breast tomosynthesis, Breast cancer, Lesion detection, Lesion classification, Medical imaging

## Abstract

**Background:**

Recent advancements in computing power and state-of-the-art algorithms have helped in more accessible and accurate diagnosis of numerous diseases. In addition, the development of de novo areas in imaging science, such as radiomics and radiogenomics, have been adding more to personalize healthcare to stratify patients better. These techniques associate imaging phenotypes with the related disease genes. Various imaging modalities have been used for years to diagnose breast cancer. Nonetheless, digital breast tomosynthesis (DBT), a state-of-the-art technique, has produced promising results comparatively. DBT, a 3D mammography, is replacing conventional 2D mammography rapidly. This technological advancement is key to AI algorithms for accurately interpreting medical images.

**Objective and methods:**

This paper presents a comprehensive review of deep learning (DL), radiomics and radiogenomics in breast image analysis. This review focuses on DBT, its extracted synthetic mammography (SM), and full-field digital mammography (FFDM). Furthermore, this survey provides systematic knowledge about DL, radiomics, and radiogenomics for beginners and advanced-level researchers.

**Results:**

A total of 500 articles were identified, with 30 studies included as the set criteria. Parallel benchmarking of radiomics, radiogenomics, and DL models applied to the DBT images could allow clinicians and researchers alike to have greater awareness as they consider clinical deployment or development of new models. This review provides a comprehensive guide to understanding the current state of early breast cancer detection using DBT images.

**Conclusion:**

Using this survey, investigators with various backgrounds can easily seek interdisciplinary science and new DL, radiomics, and radiogenomics directions towards DBT.

## Background

Breast cancer is the most prevalent type of cancer in women. In 2020, 2.3 million new cases were diagnosed, and approximately 688,000 fatalities occurred around the globe [1, 2]. It is expected that in 2023, there will be 1,958,310 new cancer cases and 609,820 cancer deaths in the United States [3]. Cancer incidence varies across countries, regions, ethnicities, and lifestyles. Regional and ethnic backgrounds can not be changed, and lifestyle and health habits are usually difficult to modify. On the other hand, the fatality rate can be reduced significantly by improving cancer detection at its early stages because it has been proven that early intervention is the most effective means to augment breast cancer survival [4].

It is very challenging to diagnose breast cancer tumors at their inception. Previous studies have suggested that knowledge deficiency, limited access to care, etc., are the major hurdles for early detection [5–7]. This lack of awareness can lead to the delayed diagnosis and treatment which can negatively impact survival rates. Furthermore, many developing countries lack diagnostic and treatment facilities for breast cancer [8]. Therefore, even if people are aware of early detection measures, they may not have access to the necessary treatment. Hence screening protocols are essential to detect subtle changes in tissue anatomy with non-invasive imaging modalities  [4]. Various imaging modalities have been developed and used to detect and diagnose a cancerous tumor in the breast at its earliest stages. However, the most common imaging modalities are: FFDM, magnetic resonance imaging (MRI) [9], 3D Ultrasound (US) [10], and Digital DBT,  [11].FFDM has the potential of improved breast cancer detection compared to film mammography, faster image acquisition, and the ability to manipulate images for better visualization[12]. On the other hand, FFDM possesses reduced spatial resolution, and the equipment cost is also high compared to film mammography[13]. Breast MRI is a useful diagnostic tool that can locate small breast lesions sometimes missed by mammography and can help detect breast cancer in women with breast implants and in younger women who tend to have dense breast tissue [14]. However, breast MRI screening results in more false positives, meaning that it can find something that turns out not to be cancer, which can result in some women getting tests and/or biopsies that are not needed [14]. False positives can be reduced with commercially available software programs in the market to enhance breast MRI scans [14]. 3D breast US can provide more detailed images of breast tissue than traditional 2D US, and it can be useful for detecting small breast lesions that may not be visible on mammography. Nonetheless, it can be more expensive than traditional 2D US, and it may take longer to perform and interpret than traditional 2D US [14].

DBT is an imaging technique that aids in the early stage detection of breast cancer. Imaging protocols used in DBT include combined FFDM and DBT, SM, dual-energy contrast-enhanced DBT (DE CE-DBT), and automated quantitative estimation of volumetric breast density. Additionally, supplemental imaging modalities such as full- and abbreviated-protocol MRI (Fp-MRI, Ab-MRI), contrast-enhanced mammography (CEM), and US can be used to improve the clinical outcomes of DBT [15–18].

DBT, on the other hand, has the potential to provide a more detailed and accurate view of breast tissue than traditional mammography. It also can reduce the need for additional imaging and biopsies. However, the downside of DBT is that it is more expensive and can expose patients to slightly more radiation than traditional mammography [14]. Diagnosing the breast cancer tumor at its inception and classifying whether the detected tumor is malignant or benign is still an open challenge.

**Fig. 1:**
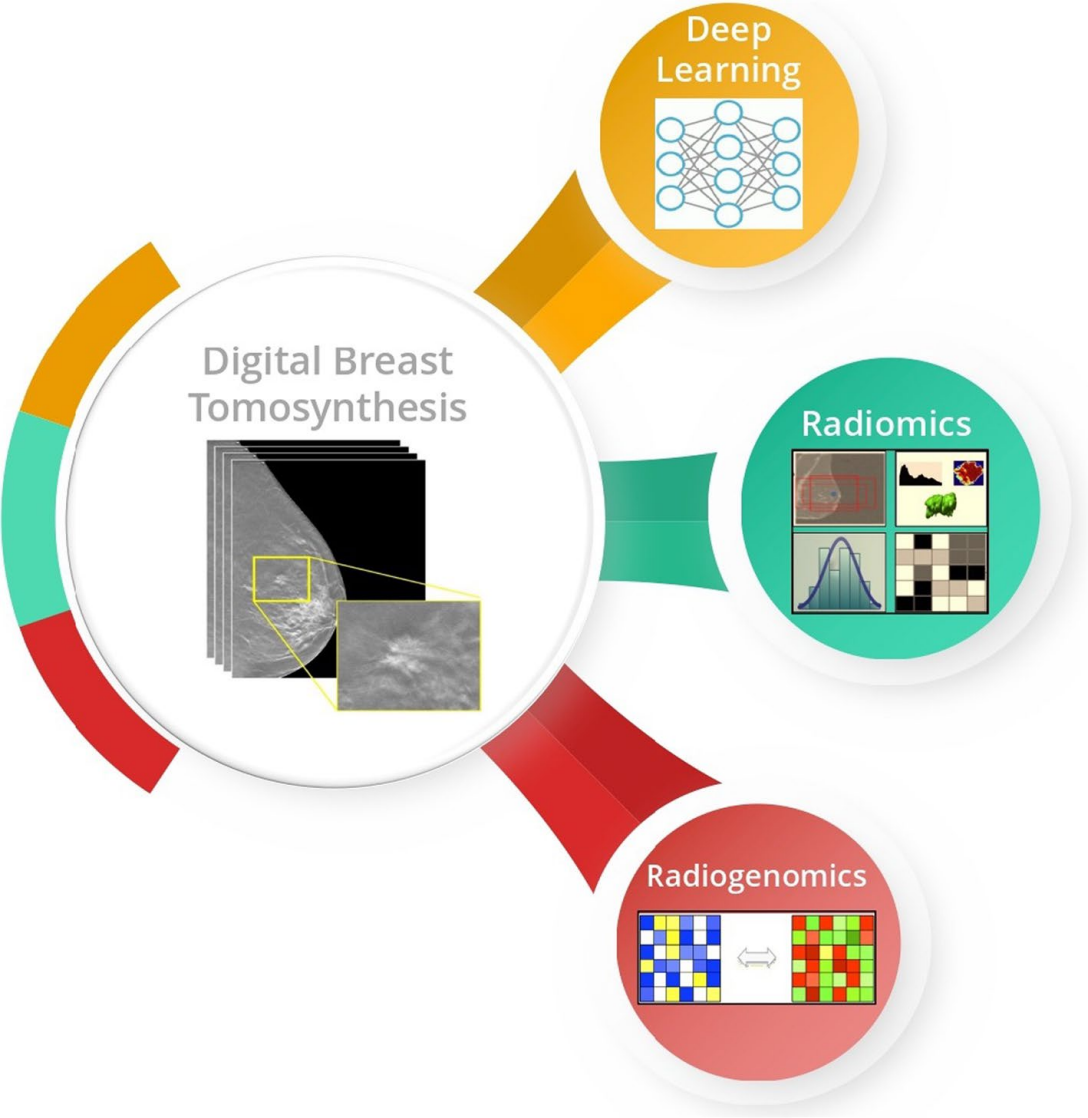
Overview of the Techniques used for DBT

For breast cancer diagnosis, state of the art technique known as DBT is used alongside FFDM. Recent findings suggest that adding imaging data, combined signatures from radiomics and genomics signatures, and the latest architectures of DL can better diagnose and stratify patients for further precise therapeutic care. A thorough literature search revealed that no prior surveys comprehensively summarize the impact of radiomics and radiogenomics for DBT images (Fig. 1). Therefore, this paper presents a systematic review of DL, radiomics, and radiogenomics applied to DBT.

DL belongs to the family of non-linear machine learning (ML) techniques. DL applied to medical images can automatically extract relevant features and learn the correlation with the target task. DL is used for breast cancer detection [19], that is, to classify an image as benign or malignant, localize any abnormality, and/or tag individual pixels as normal or abnormal. Above mentioned tasks performed by DL are commonly known as classification, object detection, and segmentation [20]. DL techniques can be used to classify breast cancer images as benign or malignant [21]. Convolutional neural networks (CNNs) have become a popular technique for analyzing medical images, including mammograms, due to their high accuracy [22]. DL techniques can detect and locate breast lesions in medical images [23]. A study used a CNN-based workflow to detect disease in PET/CT images of breast cancer patients with high sensitivity and specificity [24]. DL techniques can be used to segment breast tissue in medical images, which can detect small lesions and monitor changes in breast tissue over time [25]. Transfer learning techniques have been used to segment breast tissue in US images [21].

Radiomics is the process through which quantitative imaging features (e.g., intensity, texture) are extracted using various statistical and geometric characterization algorithms, and extracted data is used for decision support  [26]. The specific imaging features extracted by radiomics can include first-order features, such as mean, median, and standard deviation, and texture features, such as entropy, homogeneity, and contrast. Other features can include shape, size, and volume measurements, as well as features related to the intensity, gradient, and curvature of the image [14, 27–31]. Generally, several steps are taken for radiomics processing. At first, images are processed using various reconstruction algorithms, such as edge enhancement and contrast, to enhance the usability and quality of medical images. Afterwards, manual or semi-automated, or automated image segmentation is performed to identify areas of interest in 2D images (ROI) and/or volumes of interest (VOI) in 3D images. Ultimately, numeric feature extraction is carried out to obtain target characteristics of ROI or VOI [32]. Radiomics can be used for diagnosis, subtype determination, treatment response assessment, and outcome prediction in various cancers [33–36].

Radiogenomics is also known as imaging genomics, and its prime objective is to correlate imaging phenotypes with disease genes, mutations, and expression patterns. The ultimate aim of radiogenomics is to develop imaging biomarkers that associate phenotype with corresponding gene metrics for better-classifying patients for personalized therapeutic care [37]. Radiogenomics combines radiomics with genomics to develop imaging biomarkers for personalized therapeutic care. Radiogenomics aims to identify the genetic basis of imaging features and their relationship with clinical outcomes, which can help develop personalized treatment plans for cancer patients [29].

DL, radiomics, and radiogenomics are all important techniques in breast image analysis. DL can be used to identify patterns in medical images that are difficult for humans to detect [38, 39], while radiomics can extract quantitative features from medical images to develop predictive models for various clinical outcomes [39, 40]. Radiogenomics combines radiomics and genomic data to identify imaging biomarkers associated with specific genetic mutations or molecular subtypes of breast cancer [40]. Overall, these techniques can potentially improve the accuracy of breast cancer diagnosis, prognosis, and treatment planning, as well as identify new imaging biomarkers.

Parallel benchmarking of radiomics, radiogenomic, and DL models applied to the DBT images could allow clinicians and researchers alike to have greater awareness as they consider clinical deployment or development of new models. This review provides a comprehensive guide to understand the current state of personalized healthcare and early breast cancer detection using DBT images.

## Methodology and results

### Selection of papers

**Fig. 2:**
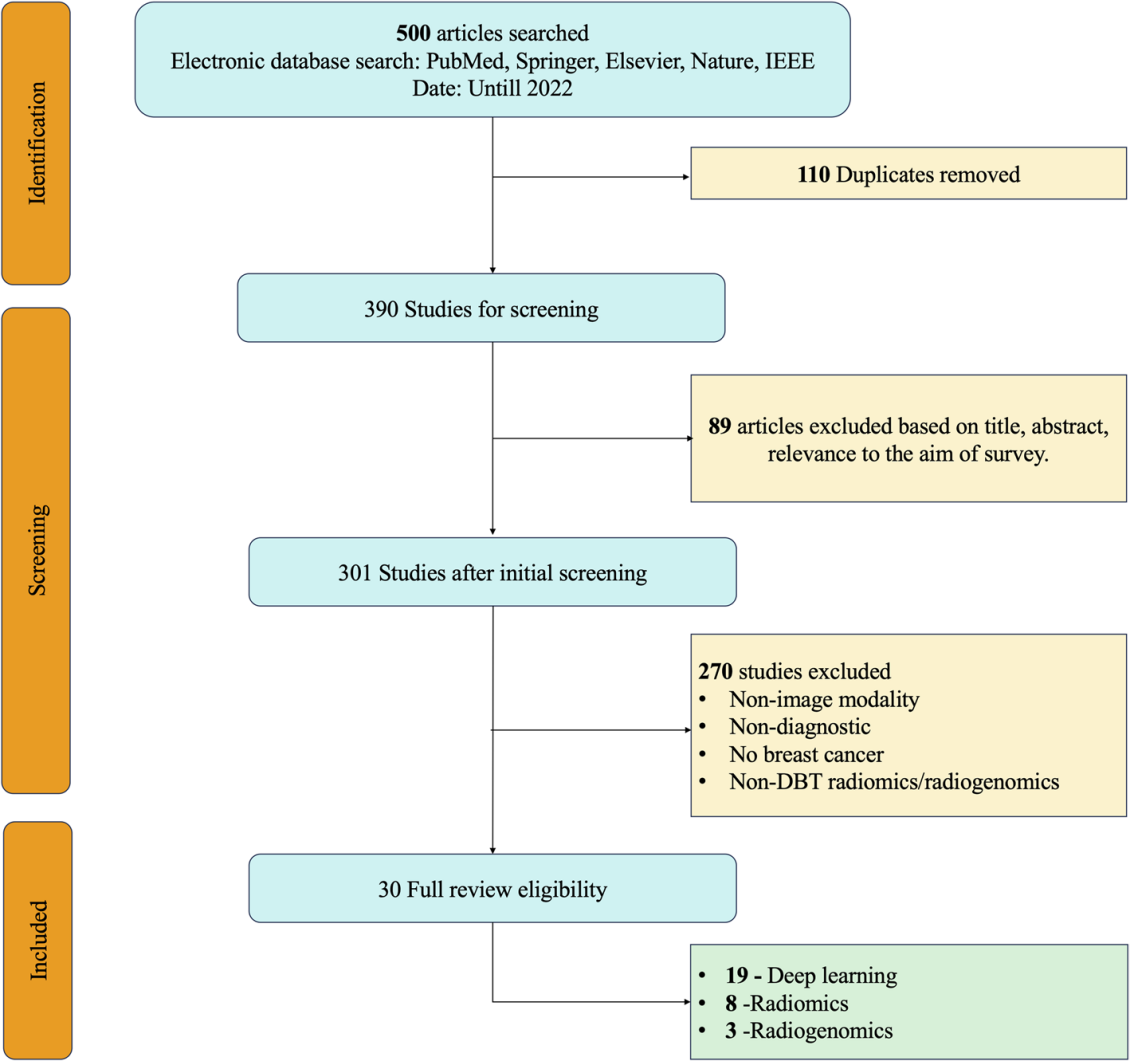
Workflow for selection of papers

Articles in this survey paper were selected from different databases, such as PubMed, ScienceDirect (Elsevier), Springer, Nature, and IEEE, that conducted studies on Digital Breast Tomosynthesis using DL, Radiomics, and Radiogenomics. We included all the studies of DL, radiomics, and radiogenomics conducted on DBT till 2022. All the studies included in this paper are in English language except for the one study that is in Chinese language. The keywords used for the selection of papers were as follows; “Digital Breast Tomosynthesis” AND “Deep Learning”, “Digital Breast Tomosynthesis” AND “Radiomics”, “Digital Breast Tomosynthesis” AND “Radiogenomics” and “Breast Cancer”. The first search generated 210 articles, the second 185, and the last 105, respectively. Papers were shortlisted based on the title, abstract, and text. We reviewed the first 210 articles on DL in DBT and selected only those used for diagnosis and localization purposes in breast cancer. We excluded other articles because they included DL for other modalities, such as breast US, thermography, MRI, positron emission tomography (PET), scintimammography, optical imaging, and computed tomography (CT). We also excluded studies that focused on non-diagnostic tasks. Furthermore, we removed papers highlighting studies of DL, radiomics, and radiogenomics related to different cancers, such as melanoma skin cancer, lung cancer, prostate cancer, colorectal cancer, and bladder cancer, among others. Among the 210 articles, we included only 20 articles that conducted studies on DL in DBT. Afterward, among the 185 articles, we included all 8 radiomics studies applied to the DBT modality, and we excluded the rest that used other modalities. Lastly, we reviewed the last 105 articles based on radiogenomics studies in DBT. After careful consideration, we found only three studies that used radiogenomics in DBT. Subsequently, all other articles that used other modalities except DBT were removed. This study has followed PRISMA checklist/flowchart method. This has been highlighted in the PRISMA diagram in Fig. 2.

## Deep learning in digital breast tomosynthesis

In this section, we discuss the DL methods used in this ever-burgeoning field of DBT for image classification, ROI detection, and segmentation for the ultimate goal of a breast cancer diagnosis. A comparison of the results is illustrated in Table 1.

### DBT image classification

In a study conducted in [41], a performance comparison was carried out between 2D and 3D mammography, trained using (CNN) with a traditional CAD algorithm that works on hand-engineered features using computation and classification methods. A total of 344 DBT reconstructions (consisting of 328 suspicious and 115 malignant soft tissue densities) were used to evaluate the detection performance. The ROI was used to measure the detection sensitivity. Researchers observed the increase of ROI sensitivity using a DL-based framework known as Caffe by Berkeley Vision and Learning Center (BVLC) [42] instead of traditional techniques; from 0.832 to 0.893 for suspicious areas of interest and from 0.852 to 0.930 for the malignant ROI.

Simuala et al. [43] proposed a hierarchical model to reduce the parameters of DCNN for the classification of tumors in DBT. Initially, they augmented 2454 mass lesions on mammograms in ROIs to 19,632 using transfer learning on a DCNN pre-trained on the ImageNet dataset. Later, features were extracted from 9120 DBTs ROIs from 228 mass lesions using DCNN pre-trained on digital mammography (DM) followed by feature selection and random forest classifier. Various parameters, such as neurons in DCNN by 87%, parameters by 34%, and multiplying and adding operations by 95% were reduced. The AUC on 89 mass lesions from 94 unique DBT cases were 0.88 and 0.9, respectively, preserving the original and truncated techniques in view.

Authors in [44] compared the performance of FFDM to DBT using DL. DBT and FFDM images of 78 biopsy-proven lesions from 76 patients were collected. In addition, FFDM, s2D, and DBT were used to obtain the ROI. For feature selection, a CNN-based pre-trained VGG19 network was used as input and SVM as a classifier. This pre-trained model was chosen because it is effective for various image recognition tasks and can be fine-tuned for specific tasks such as breast cancer diagnosis in digital breast tomosynthesis. The proposed DCNN compression approach can reduce the number of required operations by 95% while maintaining classification accuracy. SM performed best in the CC and MLO views on (ROC) perspective for lesion characterization as follows: (AUC = 0.81, SE = 0.05) and MLO view (AUC = 0.88, SE = 0.04). Regarding the soft voting used for merging CC and MLO views, DBT performed best (AUC = 0.89, SE = 0.04). Lastly, DBT significantly performed better than FFDM ($p=0.024$). Therefore, the efficacy of DBT in analyzing mass and ARD lesions is considered significant. DBT captures multiple images of the breast from different angles, which allows for the reconstruction of a 3D image of the breast tissue. This can help to reduce the effect of overlapping tissue, which can obscure small lesions in FFDM.

A model trained on FFDM was proposed in [45], which can be used for DBT. The model was based on the ResNet model. It used a 512x512 FFDM or MIP as the input image and predicted the probability of malignancy. Furthermore, initially, the conventional fine-tuning approach, the last fully connected layer, was tuned. Later, an adaptive fine-tuning system and a selected layer for optimization were used. Cross-entropy loss function and Adam optimizer were used for fine-tuning. The MIP-HM approach achieved the best (AUC = 0.847) by fine-tuning the last two layers.

The work in [46] compared FFDM vs DBT and transfer learning techniques (VGG-16) on DCNN to classify masses in breast cancer. DBT and FFDM data were collected from 441 participants, where the ROI of benign, malignant, and normal tissues were extracted for training and validation on the DCNN network. DBT vs FFDM’s classification capabilities and transfer learning validation on 2D DCNN were analyzed. The results suggest that DBT when used with FFDM, can perform best in terms of f AUC (malignant AUC = 0.917, benign AUC = 0.951, and normal AUC = 0.990) when applied to DBT images. In conclusion, DBT, along with transfer learning, outperforms FFDM. On the other hand, DBT with FFDM increases the accuracy for mass classification when trained on DCNN.

A study conducted in [47] contrived a latent bilateral feature-based method using DCNN to diagnose the masses in the DBT. Results suggest that the devised latent bilateral representation model performs better than the traditional hand-engineered features by improving the performance regarding the ROC and AUC curve. An SVM was used for the classification purpose. The avg AUC of the proposed model was 0.847 as compared to hand-engineered parts, which was 0.826.

A CAD model for mass detection in DBT was proposed in [48]. A DCNN is used for learning complex patterns in 2D slices of DBT. For the classification of mass in 2D slices, multiple instance learning (MIL) with a randomized tree is used. The performance of the devised CAD system for mass classification was much better than hand-engineered features and deep cardinality-restricted Boltzmann machines (DCaRBM). In conclusion, the proposed system achieved 86.81% accuracy, 86.6% sensitivity, and specificity of 87.5% with an AUC of 0.87 in DBT classification.

Authors in [49] proposed a CNN-based model built and optimized using transfer learning and data augmentation followed by neural network training. Ten different CNN architectures were evaluated. For data augmentation, reflection, and rotation techniques were used. Moreover, AlexNet, trained on ImageNet, was utilized for transfer learning. This model yields significant potential for classifying breast cancer on 2D and 3D mammograms. The best performance of the model for FFDM and DBT was (an AUC of 0.7274) and an (AUC of 0.6632) respectively.

A novel architecture based on a 2D CNN for the classification of DBT was proposed by  [50]. It has the potential to work with several slices as well as retain the slice-to-slice changes. A 2D pre-trained CNN was used for feature selection. For training, AlexNet trained on ImageNet was used. Different model sections were compared, such as pooling methods, feature extractors, and fusion strategies. The best performance, 0.854 auROC, was achieved by the amalgamation of AlexNet, max pooling, and late fusion.

Inspired by radiologists in clinical settings, Authors in [51] proposed a joint 2D and 3D mammography model. This model is believed to be the first-ever model (combines 2D and 3D mammograms) of its kind. The authors also believe the dataset is the largest combined dataset of 2D and 3D mammograms. The work faces the challenge of effectively using large and varying DBT data. Training a 3D CNN model with such data is computationally expensive and may lead to overfitting. To overcome this, the researchers extract fixed-size slice representations of the DBTs and employ a 2D CNN for classification, which is more computationally efficient. Initially, DBT was preprocessed; afterwards, features of DBT and DM were extracted before concatenation, and three classifiers (DBT classifier, DBT-DM classifier, and DM classifier) were used for the final classification. The model achieves 0.97 AUC, which is 34.72% more than a single imaging modality.

In work conducted by [52], a DL model was proposed to evaluate breast density for s2D using FFDM extracted from DBT. Breast density is an important factor in breast cancer screening and risk assessment. The dataset contained 78445 s2D. A ResNet-34 model was initially trained for many training samples for individual SM data. The model showed promising results close to radiologists in clinical settings with an AUC of 0.97. The proposed model has the potential to contribute to breast cancer screening and risk assessment by providing accurate breast density evaluation, which can help identify women who may benefit from additional screening or preventive measures.

Authors in [53] proposes a new reconstruction algorithm for DBT that improves image quality for both human and computer interpretation. Authors compared two reconstruction algorithms, filtered backpropagation (FBP) and FBP with iterative optimizations (EMPIRE) for detection of calcification in DBT. Subsequently, a 3D CNN was validated and tested on the data acquired by the reconstruction algorithms. The EMPIRE algorithm improves the visibility of calcification in DBT images. Also, DL has similar potential in terms of classification in calcification. Conclusively, the 3D-CNN with EMPIRE performed better than 3D-CNN with FBP (pAUC-EMPIRE = 0.880, pAUC-FBP = 8.57). The study demonstrates the potential of using DL models for improving image quality and calcification detection in DBT.

### Detection and segmentation

Apart from the classification of cancer, another prime task in evaluating medical images is the localization of cancerous mass. Cancer localization and segmentation in 3D mammography are important for accurate breast cancer diagnosis and treatment. 3D mammography provides more detailed breast tissue images than 2D mammography, which can help detect small lesions and calcifications that may be missed in 2D images. Accurate localization and segmentation of cancerous lesions can help determine the extent of cancer and guide treatment decisions, such as whether a lumpectomy or mastectomy is needed. Automated methods for breast cancer detection using 3D mammography can help reduce the workload of radiologists and improve the efficiency and accuracy of breast cancer diagnosis [50]. Numerous tumor detection and localization techniques have been devised for processing 2D and 3D medical images. However, few findings have highlighted the localization and segmentation in 3D mammography. Localizing cancer tumors in 3D mammography DBT requires expert radiologists in clinical settings to review each image. A human expert’s analysis of 3D mammograms individually is considerably time-consuming and much more costly. On the other hand, public databases for 3D mammography are very scarce. In this part of the paper, we review various models in DBT images that use object detection and segmentation techniques.

Authors in [54] developed a novel ResNet-50-based model that outperformed five out of five radiologists. Initially, patch-level classification was performed on 2D mammography on cropped images. Afterwards, an end-to-end detection model was trained on the bounding boxes, while a classification score was received from the first stage. Finally, for the classification of 2D mammography, the class probability of various bounding boxes was used using maximum suspicion projection (MSP) and 3D mammography classification. The model can detect previously negative cancer and generalize the population well with improved sensitivity of 14% and AUC of 0.945.

A faster RCNN-based CAD algorithm for mass detection in DBT is proposed in [55] and compared with DCNN-based CAD. Initially, 3D mammography z-stack images were preprocessed. Afterwards, RCNN based faster RCNN model is used for mass detection. Finally, a deep CNN model is used for the reduction in false-positive. The authors used free-response ROC(FROC) curves to compare the results between DCNN and RCNN models. RCNN-based CAD achieved an AUC = 0.96, whereas DCNN-based CAD achieved AUC = 0.92. In conclusion, RCNN based model performed better than the DCNN model.

By extending a previous study on faster RCNN on (s2D) based on DBT for segmentation and detection of tumors,  [56] proposed another 3D RCNN-based CAD model. The results of faster RCNN, 3D mask RCCN, and 2D mask RCNN on various images obtained from patients with numerous characteristics were compared to analyze the model’s efficacy for mass detection. The performance comparison between the proposed 3D mask RCNN and the other two 2D CNN CAD models were estimated on breast-based FROC curves. All three models achieved a sensitivity of 90%. The proposed model has fewer false-positives of 0.8 as compared to 2D mask RCNN with 1.24 false positives and Faster RCNN with 2.38 false positives.

A U-Net based architecture was proposed in [57] for mass segmentation in DBT in six stages: Preprocessing of DBT images, patch extraction, data augmentation, a fusion of voting scheme, mass segmentation using U-Net, and postprocessing. The model outperformed CNN, SVM, and linear discriminant analysis in terms of AUC with 0.859.

A DBT dataset was prepared and made public after annotating and curating it [58]. It consisted of 22032 reconstructed volumes of DBT extracted from 5060 patients. The dataset was divided into four parts: normal studies, additional studies without biopsy, benign studies with biopsy, and studies with a cancerous tumor. In addition, a single-phase DenseNet based model for cancerous object detection was built and tested on the dataset. This model resulted in 65% sensitivity with 2 FPs per breast.

In a study in [59] for anomaly detection, authors devised a robust method using GANs. Using a state-of-the-art GAN model, this work used normal DBT data to generate abnormal breast tissue images. Technically, the region is probably abnormal if the generated image significantly differs from the original image. Notwithstanding, the generated lesions using GAN appear irrelevant to the original ones, and the average pixel intensity in generated patches is twice the normal ones.

Authors in [60] proposed a faster-RCNN model for detecting typical architectural distortion (AD) and atypical architectural distortion in DBT. Furthermore, the spatial distribution of the mammary gland was used as base information before detecting AD. In addition, the Gabor filter and convergence maps were used to extract the glands’ distribution information. The results suggest that the model generated a sensitivity of 80% with 1.95 false positives per image.

**Table 1:** An Overview of the Deep learning Models in Digital Breast Tomosynthesis

Ref.	Model	Modality	Dataset	Results
[41]	Caffe	DBT^1^	2D mamo = 1864, 3D mamo = 339,	Mean ROI sesitivity, suspicous lesions(conventional methods = 0.8320 + $-0.040$,
			Suspicious lesions = 328, malignant lesions = 115	DL = 0.893 + $-0.003$), malignant lesions(conventional methods = 0.852 + $-0.065$,
				DL = 0.930 + $-0.046$)
[43]	AlexNet/DCNN	DM/DBT	Dataset = 2192	AUC (before pruning = 0.88, after pruning = 0.90)
[44]	VGG19	SM/DM/DBT	Dataset patients = 76, lesions = 78	AUC = 0.89 + $-0.04$ classification of malignant and benign
[45]	ResNet	DM/DBT	Patients = 62,417, exams = 198,201, images = 830,450	ROC AUC = 0.9
[46]	VGG16	DM/DBT	Patients = 441, views = 927, CC = 460, ML = 4, MLO = 463	Malignant classification (AUC = 0.91, ACC = 95.1%, SEN = 70.8%, SPE = 98.9%)
[47]	3D-DCNN	DBT	Patients = 40, reconstructed volume = 160	Avg AUC = 0.847 + $-0.012$
[48]	DCaRBM/DCNN	DBT	Images = 87, breast/volume = 87, image slices = 5040	AUC = 0.87, ACC = 86.81, SPE = 87.5, SEN = 86.6
[50]	AlexNet (2D-CNN)	DM/DBT	Data = 3705	auROC = 0.854
[49]	CNN (AlexNet)	DM/DBT	Data = 3290	auROC = 0.73
[51]	CNN (ImageNet)	DM/DBT	Patients = 1124	ACC = 0.91, F1 = 0.91, Precision = 0.93, Recall = 0.88 AUC = 0.97
[52]	ResNet-34	SM/DM/DBT	Exams = 207,776	Four class acc = 82.2, four class macro AUC = 0.95,
				Binary acc = 91.1, binary AUC = 0.971
[53]	EMPIRE/FBP	DBT	Patients = 374	pAUC = 0.880
[54]	ResNet-50	DM/DBT	Cases = 63,798	AUC = 0.95
[55]	Faster RCNN/DCNN	DBT	Cases = 89	ROC AUC = 0.96
[56]	3D-Mask-RCNN	DBT	Cases = 364	Lesion based mass detection (sensitivity = 90% with 0.8 FPs),
				Breast based mass detection (sensitivity = 90% with 0.83 FPs)
[57]	U-Net	DBT	Data = 87	SEN = 0.869, ACC = 0.871, AUC = 0.859
[58]	DenseNet	DBT	Patients = 5060, studies = 5610, DBT volumes = 22,032	Sensitivity = 65% at 2 FPS
[60]	Faster RCNN	DBT	Patients = 68, DBT volume = 265	mean true positive fraction, typical AD 0.6 + $-0.05$

## Radiomics in digital breast tomosynthesis

**Fig. 3:**
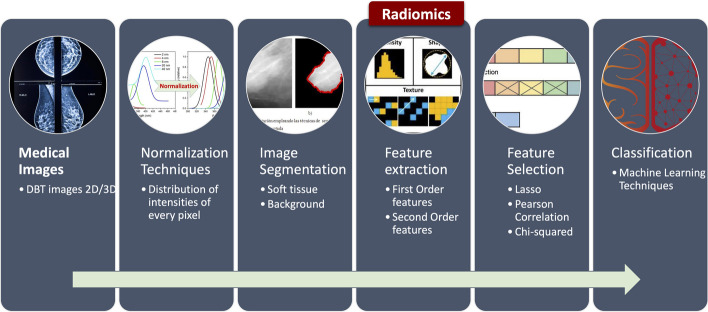
Process to implement Radiomics in digital breast tomosynthesis

Radiomics can be defined as the translation of medical images to structured numerical data, otherwise known as quantitative data. It uses a range of attributes, such as geometry, strength, and texture. These are determined from medical images to allow capturing different imaging patterns and enable phenotypical characteristics in the image for diagnosis, prognosis, prediction, decision support, monitoring, and treatment response assessment. The purpose of extracting quantifiable features from medical images lies in finding a relation between these quantitative data and biological or clinical outcomes using ML techniques [61]. The usual flow implemented to use radiomics is expressed in Fig. 3.

These techniques can be divided into two main groups: handcrafted-based and DL-based. The features-based approaches extract a set of numerical features from a segmented region. The advantages of this group are that they do not need large data sets, and they can be implemented in a short computation time. On the other hand, radiomics techniques typically use Convolutional Neural Networks to find the essential characteristics of radiological images. The main advantage of these implementations is that there is no need for image segmentation. The disadvantages of these techniques are the interpretability, the need for larger datasets, and a longer period of computational time used in their implementation [62].

In this preliminary work [63], authors used radiomics techniques in DBT in order to assess mammography-negative dense breasts. The study included 20 patients and extracted 104 features; however, only six features were selected based on MRI-based previous studies. The results revealed a correlation of three features with the tumor size: Energy, Entropy, and Dissimilarity, as well as a correlation between entropy and Estrogen Receptor Status. Moreover, skewness, entropy, and 90 percentile showed significant differences between healthy and cancer patients. The AUC obtained was 0.567.

Subsequently, the study in [64] predicted Ki-67 expression, a significant prognostic factor. Patients with low Ki-67 expression are likely to respond better to treatments. Patients diagnosed with invasive BC, 40 with low and 30 with high Ki-67 expression, were included. An open-source tool extracted 106 radiomics features, whereas the least absolute shrinkage and selection operator (LASSO) extracted 34 most discriminative features. Correlation analysis and univariate LR showed an association between selected features. Five of the 34 significant features showed the best AUC> 0.6 with a minimum p-value of 0.05. Limitations: single device images, ROIs manually segmented, limited dataset.

A model for microcalcification cluster detection in DBT with radiomics was proposed by [65]. The dataset includes 79 benign and 196 malignant cases. First, ROI was segmented. Afterwards, 170 imaging features and the 26 most significant features were selected for radiomics modeling. Since microcalcifications are shown as tiny and bright spots in DBT, shape and intensity features played an important role in the classification. The RF classifier performed best with an AUC of 0.825 among different ML classifiers. Whereas radiologists obtained AUCs = 0.840 and 0.831 when using DBT and DM. Limitations: the semi-automatic segmentation of ROIs, low population variance, and focus on one breast cancer lesion.

Authors in [66] analyzed the radiomics morphological features extraction using DBT to classify malignant and benign lesions. Furthermore, they used univariate and bivariate analyses of quantitative objective features using pattern recognition techniques and suggested a classifier for the radiologist to use in the clinical setting. The DT performed best in terms of contrast and angularity with 87.1% accuracy. Lesion texture and morphological parameters can obtain missed information of tumor characteristics for diagnostics and prognostics purposes.

A method to classify lesions on DBT images using radiomics was investigated in [67]. A public dataset of 31 malignant and 20 benign cases was used. At most, 70 various radiomics features associated with shape, existence of spicula, and texture data of lesions were extracted. Afterwards, multiple classifiers (SVM, NB, RF, and MLP) were used. SVM performed best as the benign and malignant tumor was detected with 55% and 84% accuracy, respectively. The proposed method may be helpful for a radiologist to diagnose lesions more accurately.

The objective of this study by [68] was to estimate malignancy risk among those that would be recommended for biopsy by radiologists. A dataset of 49 DBT images and 49 breast calcification images (with an average age of 51 years) classified by BI-RADS were used to build a radiomics-based classification model. The model trained on DBT achieved accuracy, sensitivity, and specificity of 0.82, 0.85, and 0.80, respectively.

A model for classifying molecular subtypes in BC radiomics features was extracted from SM that was subsequently reconstructed from DBT and was proposed in [69]. A dataset of 365 patients with invasive BC was used. Consequently, an AUC of 0.838, 0.645, and 0.556 for triple-negative TN, luminal, and HER2 subtypes were obtained from radiomics signatures, respectively. Clinical features in conjunction with radiomics features demonstrated considerably better AUC values than clinical features for identifying the triple-negative subtype.

Authors in [70] devised a method to evaluate the differential diagnosis of mass lesions in DBT. A total of 143 positive BC patients confirmed by surgery and pathology were examined between 2019 and 2020. Radiomics features based on mass lesions and the LASSO regression model were extracted. The model was built using SVM, LR, and gradient-boosting decision tree(GBDT) algorithms. Extracted lesions were 79 malignant and 65 benign. Classifiers LR, SVM, and GBDT detected optimal features as 20, 24, and 32, respectively. GBDT model achieved the best performance of AUC at 0.91.

## Radiogenomics in digital breast tomosynthesis

Radiogenomics is a newly emerged field. It has been used to associate phenotypes with the relevant disease genes. Radiogenomics is commonly known as the analysis of associations between genes of the patient and their reaction to radiation therapy [71]. Radiogenomics, most of the time, is associated with radiomics. However, radiomics, on the other hand, is the method to extract desired quantitative features using some algorithms [72]. Traditionally, the treatment is done by a one-size-fits-all approach, where the treatment is designed for the average person, this results in many side effects owing to the different human metabolisms. Contrary to this, radiogenomics’ target is to create a one-size-fits-one approach to provide personalized care to the individual patient, leading to the field of precision medicine.

Radiogenomics is a rapidly growing field and has been used for breast cancer [73], lung cancer [74], and brain cancer [75], among others. Radiogenomics in breast cancer started in 2012 when Yamamoto et al. researched the association between several genes and 26 imaging phenotypes in a small group of 10 patients on MRI [73].

Work conducted in [76] was the first study of radiogenomics in DBT to evaluate the effect of molecular subtypes on detecting BC in DBT. A total of 288 invasive DBT cases were evaluated according to the BI-RADS lexicon. Although, molecular subtypes can help detect breast cancer in DBT, the subtype is not the core parameter to determine the detection of BC. Instead, the major factors for detecting breast cancer in DBT are mass or calcification, invasive tumor size, and breast density. More extensive studies should be needed to validate these findings.

Authors in [77] evaluated the impact of prognostic factors, radiological signs, and tumor subtype on tumor size discrepancies between final histology and DBT. The study consists of 130 patients diagnosed with BC. A distinction was present if the difference between final histology and DBT was more than 5 mm. All 96 female patients and 105 cases of cancer with discrepancies were included. Conclusively, the difference in tumor size between final histology and DBT was because of the architectural distortion observed in DBT or the diagnosis of infiltrating lobular carcinomas at histology. The difference in tumor size was not affected by prognostic parameters.

Xu et al. [78] studied the relationship between X-ray signs of DBT and molecular subtypes of BC recently. The pathological data and DBT images of 153 patients with BC were evaluated. The data was divided into a triple-negative group(n33), HER-2 positive group (30), and hormone receptor (HR) positive group(90). As per BI-RADS, DBT signs of different molecular subtypes were compared. The study did not find significant differences among the groups in terms of mass, size, calcification presence, asymmetry, or architectural distortion. Conclusively, molecular subtypes of breast cancer are related to the imaging signs of DBT. Understanding these signs helps predict the molecular subtypes of breast cancer.

## Discussion

Many techniques have been used to classify cancer tumors in breast images, for instance, DL, radiomics, and radiogenomics for different modalities such as MRI, US, FFDM, and DBT. The most common DBT evaluation techniques are radiomics and DL. However, researchers have also used radiogenomics for MRI, 2D mammography, and US modalities. DL, in conjunction with radiomics, has generated promising results for breast cancer in terms of tumor classification, localization, and generation of quantitative information, personalized care, and nuclear medicine. Evaluation of breast images starts with tumor classification. Traditionally, breast cancer is diagnosed with mammography, MRI, breast US, scintimammography optical imaging, and some molecular imaging. Notwithstanding, these modalities diagnose breast cancer at a later stage. Contrary to this, DBT is a state-of-the-art modality for breast cancer detection and has been around for the last two decades. It yields promising results in an increase in cancer detection and decreases recall and false positives rates. Recent advances in AI show that it can reduce the workload of radiologists and decrease the chance of missing tumors due to human error and/or fatigue [79–82]. Various ML algorithms such as RF, LR, KNN, and SVM have been used to diagnose breast cancer using numerous imaging. Results suggest that DL outperforms traditional ML algorithms concerning a breast cancer diagnosis when the data is abundant. Research is being conducted extensively in the rapidly expanding field of computer vision, particularly in medical image analysis, to enhance existing DL methods for the classification of breast cancer in DBT.

Segmentation and detection of cancerous mass, such as solid mass or fluid-filled cysts, are considered vital tasks in the analysis of breast cancer. It is easier to segment the tumor because of the variability in benign and malignant tumors in terms of size and shape. Subsequently, the region of interest (ROI) extracts the features with a gray-level co-occurrence matrix (GLCM). Different classical, ML, and DL segmentation methods have been used to identify lesions. The standard methods are edge-based, region-based, threshold-based, unsupervised, supervised ML, U-NET, ResNet, AlexNet, and convolutional neural networks. Lately, many scientists and investigators across all modalities are using DL methods to detect and segment lesions in breast cancer due to their superiority over conventional techniques owing to their robustness and remarkable accuracy. The latest findings suggest that state-of-the-art DL techniques are becoming the backbone for breast lesions segmentation and detection and imply a further revolution shortly.

Based on the existing literature and following the trend, our perspective is that DL has shown superior performance and has been successfully applied to DBT. Initially, authors in [41] compared mammography and DBT using CNN and conventional CAD algorithms. It was observed that using DL compared to CAD increased the sensitivity of the ROI from 0.832 to 0.893 for suspicious ROIs and from 0.852 to 0.930 for malignant ROIs. A two-stage model was proposed in [43], where authors augmented mass lesions on mammograms using transfer learning on the ImageNet dataset. Afterward, transfer learning was performed from trained mammography on deep CNN to DBT. AUC before and after applying the pruning technique was 0.88 and 0.90, respectively. Furthermore, a performance comparison of FFDM and DBT using a pre-trained VGG19 network and SVM for prediction of the disease was carried out in [44]. The s2D performed best in both CC and MLO view (AUC = 0.81, SE = 0.05) and (AUC = 0.88, SE = 0.04). When CC and MLO data merged with soft voting, DBT performed significantly better (AUC = 0.89, SE = 0.04). Authors in [46] differentiated FFDM and DBT to evaluate classification capabilities and validate transfer learning on DCNN. As a result, DBT, when used with FFDM, garnered AUC (malignant AUC = 0.917, benign AUC = 0.951, and normal AUC = 0.990). It was concluded that DBT and transfer learning could outperform FFDM. In another study [47], authors devised a DCNN model to classify the masses in DBT with improved AUC to 0.847 compared to traditional methods of 0.826. A CAD model for mass detection in DBT was proposed in [48] that outperformed hand engineered method with 86.81% accuracy, 86.6% sensitivity, and specificity of 87.5% with an AUC of 0.87. Authors proposed a three-stage CNN-based model in [49], where ten different CNN architectures were evaluated. The model yielded AUC = 0.723 for FFDM and AUC = 0.66 for DBT as the best performance. The same research group  [50] extended their work and designed a 2D CNN architecture for DBT classification, which improved performance by 28.8% with auROC 0.854 compared to the 3D CNN.  [51] proposed a novel two-stage joint 2D and 3D mammogram model which improved AUC = 0.97 by 34.72% as compared to the single image modalities. In another work [52], a ResNet model for breast density detection on SM extracted from DBT was proposed. It performed as well as a trained radiologist with 0.97 AUC. Authors in [53] compared (FBP and EMPIRE) as two reconstruction algorithms for localizing calcification in DBT. 3D-CNN-EMPIRE performed well with pAUC = 0.88 compared to 3D-CNN-FBP, which is pAUC = 0.857.

Various localization and segmentation techniques have been used for breast tumor detection. A work conducted in [54] achieved a state-of-the-art performance with an increase in sensitivity by 14% and an AUC by 0.945 and outperformed radiologists. Authors in [55] proposed a novel RCNN-based CAD model that outperformed the DCNN-based CAD model with an AUC of 0.96 compared to an AUC of 0.92. By extending work, a study in [56] compared the results of faster RCNN and 2D/3D mask RCNN to analyze the efficacy of mass detection. All three models achieved 90% sensitivity. The model also demonstrated fewer false positives of 0.8 than other models. In another work [57], authors proposed a six-stage novel U-NET-based model for mass segmentation in DBT. The model outperformed SVM, CNN, and linear discriminant analysis with an AUC of 0.859. A dataset was annotated, curated, and made public by [58]. Also, a model was built and tested on the dataset, which yielded 65% sensitivity with 2 false positives per breast. A GAN-based model was proposed for anomaly detection in DBT [59]. Normal DBT scans were used to produce abnormal scans in order to use synthetic data effectively and achieve promising results. Recently, authors in [60] developed a DL-based model to detect typical and atypical (AD) in DBT and generated promising results with a sensitivity of 80% with 1.95 FPs per volume.

In addition to DL techniques, there is a rising trend to improve the detection of breast cancer in DBT with radiomics. As explained before, radiomics can be defined as the translation of medical imaging to quantitative data. The use of radiomics to detect BRCA in DBT images has been recently studied by scientists. The first study was performed four years ago. Although the results could have been more optimal, it was demonstrated that radiomics is an emerging field to improve traditional ML classifiers.

Authors in [63] conducted the first study of radiomics in DBT, resulting in a low performance with an AUC of 0.567. The reason was the scarcity of data. The study also suggested that extracted features can characterize BRCA. The study [64] extending the previous work, concluded with better accuracy; AUC > 0.6 with limitation; ROIs were segmented manually and dataset was limited. Another study [65] obtained a significantly higher performance with an AUC of 0.825 using Random Forest. However, the study focused on only one specific lesion of BRCA. Authors in [66] performed classification of BRCA lesions with an accuracy of 87.1% with a suggestion that texture and morphological features could be significant in obtaining a promising result for BRCA diagnostics and prognosis. Authors in [67] designed a model to detect a mass lesion in DBT with traditional ML algorithms, and radiomics features that resulted in an AUC of 0.91 using a GBDT. In another work [68], authors developed a model to estimate the risk of developing breast cancer using radiomics techniques that achieved an accuracy of 0.82. An approach developed regarding risk detection was proposed in [69] in which three specific subtypes of the lesion were evaluated that obtained an AUC of 0.838.

The results of the most recent radiomics study [70] show an AUC of 0.91 compared to the first approach, with an AUC of 0.567, indicating the field’s significant potential in the near future. Comparison of the results are illustrated in the Table 2.

Researchers suggest that more radiomics approaches be made since recent studies performed promising results in BRCA classification in DBT images. The number of radiomics studies is limited, and the significant features of BRCA in DBT images are not standardized.

**Table 2:** An Overview of the radiomics techniques in digital breast tomosynthesis

Ref.	Model	Modality	Dataset	Dataset	Results
			license	Size	
[63]	Linear regression	DBT	Private	Cases = 40	AUC = 0.567
[64]	Correlation analysis and ULR	DBT	Private	Women = 70	AUC = 0.698
[66]	Decision tree	CEDM/DBT	Private	Patient = 275, DBT volume = 550	View-based AUC = 0.834, case-based AUC = 0.868
[67]	SVM	DBT	Public	patient = 72, breast lesions = 93	auROC = 0.90, ACC = 87.1
[65]	Random Forest	DBT	Private	Cases = 24, lesions=51	ACC = 72.5, AUC = 0.79
[69]	Logistic Regression	DBT	Private	Patients = 49	SEN = 0.78, SPEC = 0.85, AUC = 0.80, ACC = 0.82
[68]	Ensemble Classifier	SM/DBT	Private	Patients = 365	SEN = 0.833, SPEC = 0.797, AUC = 0.838, ACC = 0.803
[70]	LR/SVM/GDBT	DBT	Private	Patients = 143, lesions = 144	ACC = 0.81, AUC = 0.91

In addition to DL and radiomics applications for DBT, a novel approach known as radiogenomics is emerging. It associates imaging phenotypes with related disease genes. The first-ever study of radiogenomics for DBT was conducted by  [76] in 2017. The effect of molecular subtypes on the detection of breast cancer in DBT was evaluated. It is suggested that molecular subtypes, presence of mass or calcification, invasive tumor size, and breast density are crucial for detecting breast cancer. In work conducted in [77], authors investigated the impact of prognostic factors, radiological signs, tumor subtype, and tumor size discrepancies between final histology and DBT. The authors suggested that the difference in tumor size between final histology and DBT is because of the architectural distortion observed on DBT or the diagnosis of infiltrating lobular carcinomas at histology. The difference in tumor size is not affected by prognostic parameters. Recently, authors in [78] investigated the relationship between X-ray signs of DBT and molecular subtypes in BC. DBT and pathological data of 153 patients with breast cancer were evaluated. It was suggested that molecular subtypes of breast cancer are related to the imaging signs of DBT. Understanding these signs helps predict the molecular subtypes of breast cancer.

As stated previously, radiomics can be defined as the extraction of quantitative information from medical images. In order to implement this, many quantitative features are extracted, such as shape, first-order, and second-order features. The selection of the most significant features is made after the extraction to go through an ML model for different purposes, such as diagnosis and prognosis. On the other hand, DL is a special artificial intelligence model that implements multi-layered artificial neural networks and has been demonstrated to perform better than traditional ML methods in medical applications. The main differences between these two methods are the sample sizes needed and the number of steps. While radiomics (a two-step process) can obtain good results with moderate sample sizes, DL (one step) uses large datasets to perform well to avoid over-fitting.

Challenges and future work:

DBT is a promising new imaging technique for breast cancer detection, offering a three-dimensional view of the breast. However, challenges remain for DL, radiomics, and radiogenomics models in analyzing DBT. One limitation is the lack of large-scale annotated datasets for DBT analysis, hindering the effectiveness of DL models [19]. The complexity of DBT images poses another challenge for extracting meaningful features in radiomics and radiogenomics models. Overfitting is a potential limitation in both DL, radiomics and radiogenomics models due to the complexity of the data and the number of features extracted. Interpreting the results is also challenging, as DL models are often considered ”black boxes,” and extracting insights from radiomics, radiogenomics models with numerous features can be difficult [83–86]. These issues can be addressed by the integration of multi-modal imaging, the development of explainable AI techniques, the incorporation of clinical data, and the validation of these models in large-scale clinical trials [87, 88]. By pursuing these avenues, it is anticipated that the accuracy and effectiveness of DBT analysis for breast cancer detection and diagnosis can be improved, leading to enhanced patient outcomes.

It has been concluded from the observed pattern that DL-based radiomics assessment may allow personalized care, commonly known as precision medicine, for the better stratification of individual patients.

## Conclusion

In this survey, we provided a systematic review of the application of DL, radiomics, and radiogenomics in the analysis of DBT. We started with the DL concepts and their state-of-the-art models applied to DBT. Furthermore, we described in detail the various DL algorithms such as; DCNN, SVM, faster RCNN, U-Net, GANs, VGG19, ResNet, and DenseNet independently and in association with traditional CAD algorithms. In a few studies, DL has outperformed human readers in diagnosing tumors in DBT. Afterwards, we presented a radiomics overview along with studies applied to DBT. We then highlighted various ML techniques applied by radiomics on DBT to extract different patterns that are subsequently used for diagnosis and prognosis. The ML techniques we highlighted for extracting radiomics features in our survey are DT, SVM, EC, Logistic Regression, Linear Regression, RF and correlation analysis, and univariate linear regression. Radiomics is generating promising results, although it requires improvement. Lastly, we reviewed the radiogenomics studies conducted on DBT and how it could be used for stratifying the patients. All these techniques can improve the diagnosis, prognosis, and prediction to provide better-personalized care for individuals.

## Data Availability

All papers are available on publisher websites. All data generated or analyzed during this study are included in this published article.

## Abbreviations

FFDM: Full-field digital mammography
MRI: Magnetic resonance imaging
PET: Positron emission tomography
CT: Computed tomography
US: Ultrasound
DBT: Digital breast tomosynthesis
DL: Deep learning
ROI: Region of interest
VOI: Volumes of interest
CAD: Computer aided diagnosis
DM: Digital mammography
CC: Craniocaudal
MLO: Mediolateral oblique
MIP: Maximum intensity projection
SM: Synthetic mammography
